# Statin treatment can reduce incidence of early seizure in acute ischemic stroke: A propensity score analysis

**DOI:** 10.1038/s41598-020-58652-w

**Published:** 2020-02-06

**Authors:** Soichiro Matsubara, Tomotaka Tanaka, Shinya Tomari, Kazuki Fukuma, Hiroyuki Ishiyama, Soichiro Abe, Takuro Arimizu, Yoshitaka Yamaguchi, Soshiro Ogata, Kunihiro Nishimura, Masatoshi Koga, Yukio Ando, Kazunori Toyoda, Masafumi Ihara

**Affiliations:** 1 Department of Cerebrovascular Medicine, National Cerebral and Cardiovascular Center, Suita, Osaka, Japan; 2 Department of Neurology, National Cerebral and Cardiovascular Center, Suita, Osaka, Japan; 3 Departments of Preventive Medicine and Epidemiology, National Cerebral and Cardiovascular Center, Suita, Osaka, Japan; 4 Department of Neurology, Graduate School of Medical Sciences, Kumamoto University, Kumamoto, Japan

**Keywords:** Stroke, Epilepsy

## Abstract

A previous study showed early statin administration in patients with acute ischemic stroke (AIS) was associated with a lower risk of early-onset seizure (ES), which is a high risk of epilepsy, but this retrospective study design may not have eliminated confounding factor effects. We aimed to verify the determinants and prognostic significance of ES and clarify the effects of statin administration. Consecutive AIS patients without a history of epilepsy were enrolled. The relationship between ES (within 7 days of index-stroke) and statin treatment was assessed using multivariate and propensity scores (PS). Of 2,969 patients with AIS, 1,623 (54.6%) were treated with statin, and 66 (2.2%) developed ES. In logistic regression models, cortical stroke lesion [odds ratio (OR), 2.82; 95% confidence interval (CI), 1.29–7.28) and pre-morbid modified Rankin Scale (per 1 point) (OR, 1.39; 95% CI, 1.18–1.65) were higher risks for ES, while statin significantly reduced the risk of ES (OR, 0.44; 95% CI, 0.24–0.79). In accordance with PS-matching, statin treatment produced consistent results for ES after adjusting by inverse probability of treatment-weighting PS (OR, 0.41; 95% CI, 0.22–0.75). In conclusion, as previously, statin treatment was independently associated with a lower risk of ES in AIS.

## Introduction

Over the last decade, there have been notable improvements in the treatment of acute stroke. While the number of patients surviving stroke is expected to increase, optimal management of post-stroke patients remains problematic^1^. Stroke is the most common comorbidity in epilepsy in elderly people^2^. Recent reviews have reported acute symptomatic seizure in acute ischemic stroke (AIS), so called “early-onset seizure” (ES), was a risk of post-stroke epilepsy (PSE)^3–5^. Estimates of the rate of ES in patients with an AIS in the last decade range from 2% to 6.5%^5,6^. Recently, the predicting score of PSE with AIS was published, with ES the most significant risk factor^7^.

The lack of robust evidence in previous studies has meant prophylactic use of anti-epileptic drugs (AED) for ES or PSE remains controversial; indeed, current stroke guidelines do not recommend use of AED for such purposes^8,9^. Statin is an inhibitor of 3-hydroxy-3-methyl glutaryl coenzyme A (HMG-CoA) reductase and a key drug in the management of acute phase or for the prevention of atherosclerotic diseases, including stroke and coronary artery disease, and is thought to possess neuroprotective properties^10^. Statin administration can lead to the modification of epileptogenic processes, presumably from its AED effects^11–16^. Early use of statins reduced the risk of ES in the management of AIS, and was associated with a lower risk of progression of ES into PSE^11^; however, the retrospective design and small number of participants in the study meant confounding factors may not have been eliminated. For example, cardioembolic strokes occur in cortical areas more frequently and are treated with statin less frequently in the acute phase. Stroke subtypes should therefore be acknowledged in any retrospective analysis.

There is a paucity of clinical evidence on the association between statin and seizure. We therefore aimed to verify the predisposing factors in ES in patients with AIS from our observational data, as well as clarify the association between statin administration and ES using propensity score (PS) methods.

## Methods

### Study population

All patients admitted to the Department of Stroke and Cerebrovascular Diseases of the National Cerebral and Cardiovascular Center were registered in a database (Clinical Trials.gov: NCT02251665). We identified consecutive patients with AIS admitted within 3 days of onset between August 2012 and July 2016. AIS was defined as the acute onset of focal neurological symptoms lasting 24 h or longer and confirmed by MRI. The following patients were excluded: (i) those diagnosed with epilepsy before index-stroke; (ii) those with stroke due to a trauma, intracerebral hemorrhage, or subarachnoid hemorrhage; (iii) those diagnosed with transient ischemic attack after admission; and (iv) those who did not undergo MRI.

The present study was approved by the Institutional Ethical Committee of the National Cerebral and Cardiovascular Center and conducted in accordance with relevant institutional guidelines. The ethics committee granted a waiver to conduct this study without written informed consent.

### Data collection and definitions

The collected data included information regarding patient clinical history and presentation, laboratory, imaging, electroencephalographic, treatment, and outcomes [modified Rankin Scale (mRS) score and mortality] at discharge. The data collectors were unaware of the current study.

Stroke severity was measured using the National Institutes of Health Stroke Scale (NIHSS) score on hospital admission, and trichotomized into three levels (1, NIHSS < 9; 2, NIHSS 9–15; 3, NIHSS > 15)^17^. We classified stroke subtypes according to the Trial of Org 10172 in Acute Stroke Treatment (TOAST) classification^18^. We estimated vascular territory according to a published atlas^19^, and the AIS lesions according to the Alberata Stroke Program Early CT Stroke score for MRI (DWI-ASPECT)^20^.

In the current study, patients were recruited if any occurrence of seizure had occurred, after careful evaluation by a trained neurologist(s). ES was defined as a seizure within 7 days of stroke^21^. We assessed seizure semiology before treatment, the deterioration of consciousness, and any subtle neurological manifestations in the stroke care unit or stroke ward. An imaging study was performed to exclude recurrence of stroke. Electroencephalography (EEG) was also performed if patients previously had, or were deemed likely to have, seizure. The EEG findings were obtained by two trained neurologists in neurophysiology (S.M., T.T.).

### Clinical management

All patients were undergoing treatment for strokes, including antiplatelet agents, anticoagulants, and edaravone as a neuroprotectant, with some having endovascular treatment or decompressive craniectomy, in line with current guidelines^8^. Statins were used in patients whose stroke was presumed to be of atherosclerotic origin, including small-vessel occlusion and large-artery atherosclerosis, with or without consideration of the low-density lipoprotein cholesterol level, and not for the prevention of seizure^8^. Statin use was classified into two phases: before stroke or in the acute phase. Statin treatment in acute phase was defined as starting within 3 days of index-stroke onset. Types of statin included pitavastatin, atorvastatin, simvastatin, rosuvastatin and pravastatin. AEDs were administered according to the current guidelines on seizures^22^ if patients needed treatment, regardless of EEG findings.

### Statistical analysis

We examined the differences between the groups using Pearson’s chi-square test and Fisher’s exact test for categorical data, and Welch’s t test and Wilcoxon’s rank sum test for continuous variables. All P-values were 2-sided, with P-values of <0.05 considered statistically significant.

Firstly, logistic regression was used for multivariate analysis of clinical characteristics pertaining to ES, and the associations between ES and clinical outcomes. Pre-specified variables determined to be clinically important were also included in the model.

Secondly, PS were estimated using a logistic regression model. Possible confounders were chosen for their potential association with statin on clinical knowledge. The predicted probability of preprocedural statins was calculated by fitting a logistic regression model, using all clinically relevant variables. PS was used with PS-matching and inverse-probability-of-treatment weighted (IPTW) methods^23^. ES and outcome at discharge were analyzed as dependent variables. In PS-matching methods, rigorous adjustment was performed with PS matching using the following algorithm: 1:1 optimal match with caliper width 1/5 logit of the SD and no replacement^24^. In the IPTW method, patients were weighted by the inverse probability of receiving statin, the average treatment effect (ATE) and average treatment effect on the untreated (ATT) were estimated by computing the difference between the observed and potential outcomes resulting from the presence and absence of statin treatment for each subject.

Two-hundred sixteen subjects with missing values were excluded from the multivariate and PS analysis. Statistical analysis was performed using R version 3.5.0 (R Core Team (2018). R: A language and environment for statistical computing. R Foundation for Statistical Computing, Vienna, Austria. URL https://www.R-project.org/.).

## Results

### General characteristics

We identified 4595 patients with acute stroke, including intracranial hemorrhage, transient ischemic attack, and 2969 patients with AIS. Of the patients with AIS (1144 males, mean age: 74 ± 12 years old), 1623 (54.6%) were treated with statin (93 only before index-stroke and 1530 in acute phase after admission), and 66 (2.2%) developed ES during the hospitalization. Comparison of the baseline characteristics between the patients with ES (ES group) and those without ES (non-ES group) are shown in Table 1. Compared with the non-ES group, the ES group were older, more dependent (higher mRS), and less likely to have dyslipidemia and higher NIHSS. The C-reactive protein level and white blood cell count were higher in the ES than non-ES group. Cardioembolism was the most common etiology of AIS and was higher for the ES than non-ES group, while small-vessel occlusion was lower for the ES than non-ES group. Regarding imaging characteristics, the ES group had cortical stroke lesion and large vessel stenosis more frequently than the non-ES group.

**Table 1:** Baseline characteristics of the study subjects.

	ES group (n = 66)	Non-ES group (n = 2903)	P value
Background			
Age, years	78.4 ± 9.8	74.1 ± 12.4	0.005
Sex, male	36 (55)	1789 (62)	0.252
Body mass index, kg/m^2^	20.8 ± 3.7	22.5 ± 3.8	<0.001
Pre-morbid mRS, point	2.5 [0–4]	0 [0–2]	<0.001
Hypertension	46 (70)	2279 (79)	0.096
Dyslipidemia	24 (36)	1536 (53)	0.009
Diabetes mellitus	19 (29)	735 (25)	0.567
Atrial fibrillation	21 (32)	810 (28)	0.489
Past history of stroke	27 (41)	902 (31)	0.106
Congestive heart failure	12 (18)	349 (12)	0.129
Current smoking	9 (14)	551 (19)	0.220
Current alcohol consumption	13 (20)	1146 (39)	<0.001
Laboratory data			
LDL-cholesterol, mg/dl	106.9 ± 38.6	114.9 ± 35.3	0.069
HDL-cholesterol, mg/dl	50.4 ± 14.4	50.5 ± 14.2	0.933
Total-cholesterol, mg/dl	181.9 ± 43.7	190.3 ± 41.1	0.100
Fasting plasma glucose, mg/dl	147.6 ± 70.5	136.1 ± 51.6	0.076
BUN, mg/dl	19.8 ± 7.8	18.7 ± 9.0	0.324
Creatinine, mg/dl	1.2 ± 1.0	1.1 ± 1.0	0.409
C-reactive protein, mg/dl	1.4 ± 3.8	0.7 ± 2.1	0.018
White blood cell count, x10^3^/μl	8.3 ± 4.0	7.2 ± 2.6	<0.001
Clinical characteristics			
Systolic blood pressure, mm Hg	153.3 ± 32.6	160.9 ± 38.1	0.109
Diastolic blood pressure, mm Hg	88.1 ± 24.6	85.5 ± 20.0	0.396
Initial NIHSS, point	12.5 [5–25.3]	4 [2–11]	<0.001
NIHSS severity			<0.001
1, NIHSS ≤ 8	24 (36)	2043 (70)	
2, 9 ≤ NIHSS ≤ 15	15 (23)	296 (10)	
3, 16 ≤ NIHSS	27 (41)	591 (19)	
Classification of subtype of AIS (TOAST)			<0.001
Small-vessel occlusion	1 (1.5)	746 (26)	<0.001
Large-artery atherosclerosis	12 (18)	453 (16)	0.606
Cardioembolism	32 (49)	970 (33)	0.012
Imaging characteristics			
DWI-ASPECTS, point	10 [7–10]	10 [9,10]	0.055
Hemorrhagic stroke	10 (15)	461 (16)	1.000
Symptomatic hemorrhagic stroke	1 (1.5)	51 (1.8)	1.000
Cortical stroke lesion	59 (89)	1598 (55)	<0.001
Cortical stroke lesion in the MCA territory	54 (82)	1321 (46)	<0.001
Cortical stroke lesion in the ACA territory	12 (18)	293 (10)	0.040
Cortical stroke lesion in the PCA territory	10 (15)	334 (11)	0.333
Large vessel occlusion or stenosis	33 (50)	1458 (50)	1.000
Large vessel occlusion	27 (38)	930 (32)	0.309
Large vessel stenosis	8 (11)	629 (22)	0.029

Data are presented as mean ± standard deviation, median [interquartile range] or absolute (percentage) values. ES, early seizure; mRS, modified Rankin Scale; LDL, low-density cholesterol; HDL, high-density cholesterol; BUN, blood urea nitrogen; NIHSS, National Institutes of Health Stroke Scale; AIS, acute ischemic stroke; TOAST, Trial of Org 10172 in Acute Stroke Treatment; DWI-ASPECTS, Alberata Stroke Program Early CT Stroke score for MRI; MCA, middle cerebral artery; ACA, anterior cerebral artery; PCA, posterior cerebral artery.

### Seizure and EEG profile

Among the 66 patients with ES, 28 (42.4%) had a focal aware seizure, 21 (31.8%) a focal impaired awareness seizure, and 27 (40.9%) a secondary generalized convulsion. Of those with ES, 22 (33.3%) developed status epilepticus. The majority (48 of 66; 72.7%) of all ES occurred during the first 24 hours, 12 (18.2%) occurred during 24 to 48 hours, and 6 (9.1%) occurred 3–7 days after index-stroke. Of 66 patients with ES, 57 (86.3%) had EEG and 20 (30.3%) showed epileptiform discharges. Nine patients with ES did not undergo EEG examination due to the following reasons: the seizure episode taking place out-of-hours for EEG examination in four, too critical medical condition to be eligible for EEG examination in three, and unknown reasons in two.

### Treatment and Outcomes at discharge

Comparison of treatment and outcomes at discharge between the ES and non-ES groups are shown in Table 2. There were no differences among the two groups regarding intravenous alteplase, endovascular therapy and edaravone. The ES group received statin less frequently, especially in the acute phase, compared to non-ES group. There were no differences in the timing of statin use (before or after index-stroke) among the two groups. Patients with ES had higher mRS scores than those without ES. Of the 66 patients with ES, 54 were started taking AEDs from the onset of seizure, and 28 patients were them continued at discharge.

**Table 2:** Treatment and outcomes at discharge of the study subjects.

	ES group (n = 66)	Non-ES group (n = 2903)	P value
Intravenous alteplase	9 (14)	421 (15)	1.000
Endovascular therapy	5 (10)	183 (7.3)	0.402
Edaravone	40 (61)	1873 (65)	0.517
Statin treatment	20 (30)	1603 (55)	<0.001
Statin use before index-stroke	11 (17)	764 (26)	0.089
Statin use in acute phase	19 (29)	1511 (52)	<0.001
Statin treatment after acute phase	22 (33)	1673 (58)	<0.001
mRS scores at discharge	4 [3–5]	2 [1–4]	0.009
Hospital stay, day	25.5 [18–33.5]	18 [12–27]	<0.001
In hospital mortality	3 (4.6)	73 (2.5)	0.238

Data are presented as median [interquartile range] or absolute (percentage) values. mRS, modified Rankin Scale.

### Multivariate analysis for ES and outcomes at discharge

**Figure 1:**
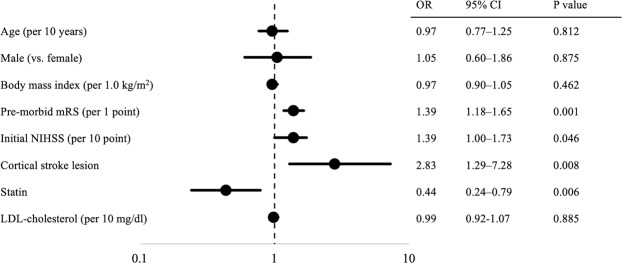
Adjusted odds ratio for each variable as to ES in multivariate analysis. mRS, modified Rankin Scale; NIHSS, National Institutes of Health Stroke Scale; OR, odds ratio; CI confidence interval; LDL, low-density cholesterol.

In logistic regression models adjusted by age, sex, body mass index (BMI), pre-morbid mRS, initial NIHSS, LDL-cholesterol level, subtypes of stroke (TOAST classification), cortical stroke lesion, and statin treatment, cortical stroke lesion [odds ratio (OR), 2.83; 95% confidence interval (CI), 1.29–7.28; P = 0.008), pre-morbid mRS (per 1 point) (OR, 1.39; 95% CI, 1.18–1.65; P = 0.001), and initial NIHSS (per 10 point) (OR, 1.39; 95% CI, 1.00–1.73; P = 0.046) were higher risks of ES, while statin treatment significantly reduced the risk of ES (OR, 0.44; 95% CI, 0.24–0.79; P = 0.006) (Fig. 1).

After adjusting for age, sex, BMI, pre-morbid mRS, initial NIHSS, and DWI-ASPECTS, ES was not independently associated with poor functional outcomes (an mRS score of 4-5: OR, 1.29; 95% CI, 0.68–2.49) or mortality (OR, 0.79; 95% CI, 0.18–2.58) at discharge.

### Propensity score analysis

**Figure 2:**
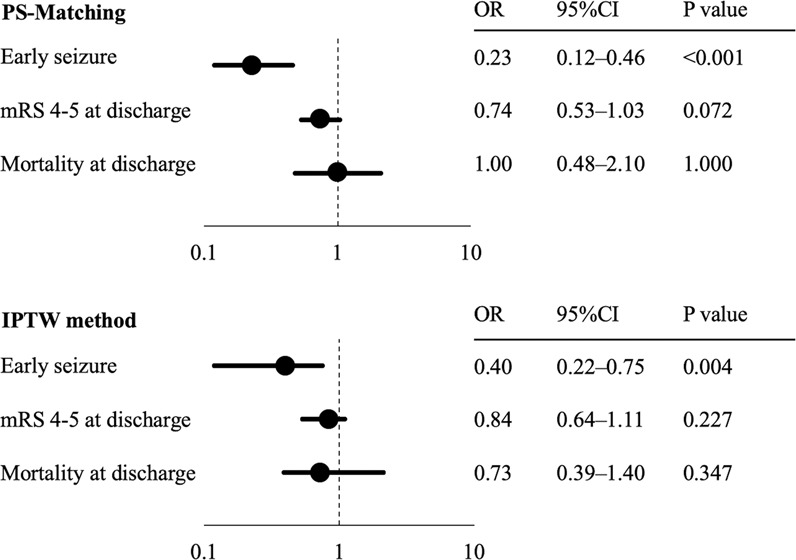
Clinical significances of statin treatment for ES and outcome at discharge in PS methods. PS, propensity score; IPTW, inverse-probability-of-treatment weighted; OR, odds ratio; CI confidence interval; mRS, modified Rankin Score.

Propensity score (PS) precision was evaluated using a receiver operating characteristic curve, with the area under the curve showing good precision equaling 0.730 (Supplemental Figure). Of patients who received statin, 54.6% (n = 886) were matched to similar patients who did not. The covariate balance in the PS-matched patients was enormously improved (Table 3). When compared to no use, statin reduced the rate of ES significantly by both the PS-matching (OR, 0.23; 95% CI, 0.12–0.46; P < 0.001) and IPTW method (OR 0.40; 95% CI 0.22–0.75; P = 0.004) (Fig. 2). Regarding outcome at discharge, poor functional outcome (mRS 4-5) tended to be decreased in patients with statin by both the PS method and IPTW method, but not significantly (PS-matching: OR, 0.74; 95% CI, 0.52–1.03; P = 0.072; IPTW: OR, 0.84; 95% CI, 0.64–1.11; P = 0.227; respectively). In the IPTW method, ATE, representing absolute risk difference of ES in the whole cohort, was 1.9% (statin 1.3% vs. no use 3.2%, P < 0.001), and ATT, representing absolute risk difference of ES in untreated patients, was 1.7% (statin use 1.2% vs. no statin use 2.9%, P = 0.002).

**Table 3:** Patients characteristics of the study subjects before and after PS-matching.

Variable	Before PS-matching			After PS-matching		
	statin (n = 1623)	no-use (n = 1345)	SMD*	statin (n = 886)	no-use (n = 886)	SMD*
Age, years	72.9 ± 11.2	75.8 ± 13.4	0.250	74.0 ± 10.9	74.5 ± 13.5	0.047
Sex, male	964 (63)	713 (60)	0.068	542 (61)	546 (62)	0.042
Body mass index, kg/m^2^	23.1 ± 3.8	21.7 ± 3.7	0.345	22.4 ± 3.7	22.3 ± 3.7	0.022
Pre-morbid mRS	0 [0–1]	0 [0–2]	0.202	0 [0–2]	0 [0–1]	0.012
Atrial fibrillation	327 (20)	504 (38)	0.411	255 (29)	244 (28)	0.027
Past history of stroke	537 (33)	392 (29)	0.077	288 (33)	279 (32)	0.022
Current smoking	346 (21)	214 (16)	0.137	143 (16)	157 (18)	0.043
Current alcohol consumption	671 (41)	484 (36)	0.108	334 (38)	339 (38)	0.011
Systolic blood pressure, mm Hg	163.1 ± 28.9	157.9 ± 46.5	0.210	158.9 ± 26.8	159.3 ± 27.7	0.014
LDL-cholesterol, mg/dl	121.8 ± 37.0	106.1 ± 31.4	0.399	111.9 ± 34.3	111.6 ± 31.5	0.073
Fasting plasma glucose, mg/dl	139.9 ± 56.0	132.0 ± 46.7	0.146	135.8 ± 52.5	134.0 ± 49.2	0.006
C-reactive protein, mg/dl	0.56 ± 1.73	0.97 ± 2.59	0.207	0.71 ± 2.06	0.63 ± 1.74	0.036
Stroke subtype			0.615			0.056
Small-vessel occlusion	504 (31)	243 (19)		204 (23)	222 (25)	
Large-artery atherosclerosis	369 (22)	111 (8)		98 (11)	101 (11)	
Cardioembolism	369 (23)	633 (47)		312 (35)	305 (34)	
Initial NIHSS score, point	3 [1–7]	6 [2–18]	0.556	4 [2–10]	4 [2–10]	0.009
DWI-ASPECTS, point	10 [8–10]	10 [9,10]	0.289	10 [9,10]	10 [9,10]	0.026
Cortical stroke lesion	807 (50)	849 (63)	0.239	492 (55)	489 (55)	0.007
Large vessel stenosis	446 (27)	191 (14)	0.286	171 (19)	159 (18)	0.034

Data are presented as mean ± standard deviation, median [interquartile range] or absolute (percentage) value. PS, propensity score; mRS, modified Rankin Scale; LDL, low-density lipoprotein; DWI-ASPECTS, Alberata Stroke Program Early CT Stroke score for MRI. *A standardized mean difference (SMD) of ≤0.1 indicates a negligible difference in the measured variables between groups.

## Discussion

The major findings of this study were as follows: (i) cortical stroke lesion, pre-morbid mRS, and initial NIHSS were higher risks for ES, while statin treatment was significantly associated with a lower risk for ES in patients with AIS, and (ii) statin treatment still had a consistent result after adjusting by PS methods.

Several previous studies have reported AIS patients with cortical stroke lesion, higher stroke severity or other comorbidities had acute symptomatic seizures more frequently than those without^11,25,26^. We found that cortical stroke lesion, higher NIHSS and higher degree of disability, represented by pre-morbid mRS, were independently associated with ES in the study subjects. Our study confirmed these factors also promote ES after AIS.

Recent studies have shown that statin was associated with reduced risk of ES^11^ and PSE^11,15,16^ after stroke. Statin was also associated with a lower risk of epilepsy in the absence of stroke^12–14^. Our study demonstrates early use of statin reduced ES in two PS analyses, adjusted by baseline characteristics, stroke severity, stroke subtypes, and lipid profile. These findings suggest statin is independently associated with lower incidence of ES than other factors. Using these two methods, confounding factors could be reduced or eliminated by measured covariates^23^. The effect size of statin was almost equivalent to that found in previous studies^11,15,16^. The current study, however, differs from previous analyses by reducing selection biases through the addition of PS analyses, adding robustness regarding the inhibitory effect of statin for ES. Although the rate of ES was relatively low compared to the other ES study^5,6,11^, it is intriguing that the effect of statin, represented by ATT or ATE, was estimated at 1.9% and 1.7%, respectively.

ES is considered a transient metabolic and biochemical phenomenon, incorporating hypoxia, metabolic dysfunction, global hypoperfusion, hyperperfusion, glutamate excitotoxicity, ion channel dysfunction, and blood-brain barrier disruption in acute phase^4,5^. ES was indicated to be a significant risk factor for PSE in a meta-analysis^3^. Experimental studies have provided convincing evidence that statins have a neuroprotective, and anti-seizure, effect. It has been reported that lipophilic statins, such as atorvastatin, pitavastatin and simvastatin, but also hydrophilic statins, including simvastatin, have a suppressive effect on seizure development in an experimental animal model^27–30^. The possible anti-seizure mechanism of statins may be related to the reduction in neuroinflammation mediated by a decrease in glutamate, pro-inflammatory cytokines (such as interleukin-6, interleukin-10, tumor necrosis factor alpha, and interferon-γ) and action in the nitrergic system^30^. In accordance with our findings and a previous study^11^, early use of statin was independently associated with a lower incidence of ES, which may be related to one or more of the mechanisms described above.

Regardless of stroke subtype or cholesterol levels, our study suggested that statin imparted a protective effect against ES; however, the risk of statin-induced hemorrhage must also be considered^31,32^. A post-hoc analysis of randomized clinical trials showed patients with ischemic stroke of small-vessel subtypes and treated by high dose of atorvastatin had a higher incidence of hemorrhagic stroke, though hemorrhage risk was not increased in patients with stroke of a large-artery atherosclerosis or cardioembolic origin^31^. In addition, active statin therapy was not associated with significant increase in ICH, and had a significant reduction in all stroke and all-cause mortality in a meta-analysis of 31 randomized controlled trials of statin therapy^32^. Therefore, future prospective studies testing the effect of statins on early seizure after stroke should be considered with such factors in mind.

We acknowledge some limitations in this study. Firstly, the study was performed in a single-center, with retrospective design, and its observation period was short (only comprising hospital stay). Although the 165 patients were initiated statin after acute phase, the reasons behind why they were not initiated within 3 days could not be found. We also excluded subjects with missing data from the analysis and could not include all potential factors not to start statin, i.e. dysphasia, drug hypersensitivity, progression or recurrence of stroke, infection, etc. Such factors may have caused selection bias and limited generalizability. Secondly, acute statin treatment for stroke is not well-established, and our management of AIS could not be sufficiently aligned to other stroke guidelines, further limiting generalizability. Thirdly, there was potential for misdiagnosis regarding limb-shaking TIA for patients with severe carotid stenosis or occlusion; however, this is a rare phenomenon in carotid disease, and with MRI and EEG assessment by a professional neurologist, the risk of misdiagnosis should be minimized. Fourthly, we could not assess doses or types of statins because of the difficulty in acquiring subjects in each group. The neuroprotective effects of statins likely changed according to different doses and type of drug, for example. The study, however, did demonstrate early use of statins was significantly beneficial, including in low doses of commonly-prescribed types. We hope future prospective studies will be performed to address some of the limitations of the current study.

In conclusion, using PS analysis, this study showed robustly that statin treatment can be associated with a lower risk of ES in AIS.

## Supplementary information

Online Supplemental Material.

## Data Availability

The data supporting this study are available from the corresponding author upon request.
