# Supplementary material for: Statin treatment can reduce incidence of early seizure in acute ischemic stroke: A propensity score analysis
Source: Sci Rep. 2020 Feb 6;10:1968. doi: 10.1038/s41598-020-58652-w (PMC7005175; doi:10.1038/s41598-020-58652-w)

ONLINE SUPPLEMENTAL MATERIAL

Statin treatment can reduce incidence of early seizure in acute ischemic stroke: A propensity score analysis.

*Soichiro Matsubara[1,4], Tomotaka Tanaka[2], Shinya Tomari[1], Kazuki Fukuma[2], Hiroyuki Ishiyama[2], Soichiro Abe[2], Takuro Arimizu[1], Yoshitaka Yamaguchi[1], Soshiro Ogata[3], Kunihiro Nishimura[3], Masatoshi Koga[1], Yukio Ando[4], Kazunori Toyoda[1,] Masafumi Ihara[2]

[1]Department of Cerebrovascular Medicine, National Cerebral and Cardiovascular Center, Suita, Osaka, Japan.

[2]Department of Neurology, National Cerebral and Cardiovascular Center, Suita, Osaka, Japan.

[3]Departments of Preventive Medicine and Epidemiology, National Cerebral and Cardiovascular Center, Suita, Osaka, Japan.

[4]Department of Neurology, Graduate School of Medical Sciences, Kumamoto University, Kumamoto, Japan

Correspondence: Soichiro Matsubara, MD, PhD

Department of Cerebrovascular Medicine, National Cerebral and Cardiovascular Center, Suita, Japan

5-7-1 Fujishirodai, Suita, Osaka 565-8565, Japan

E-mail: matsubara-soichiro@umin.ac.jp

Phone: +81-6-6833-5012; Fax: +81-6-6872-7468

Supplemental Figure. Receiver operating characteristic (ROC) curve showing the prognostic value of the propensity score for statin treatment. ROC curve was calculated by fitting a logistic regression model, using all clinically relevant variables, as shown in Table 3. The area under the curve is 0.730 (95% confidence interval, 0.598–0.740)

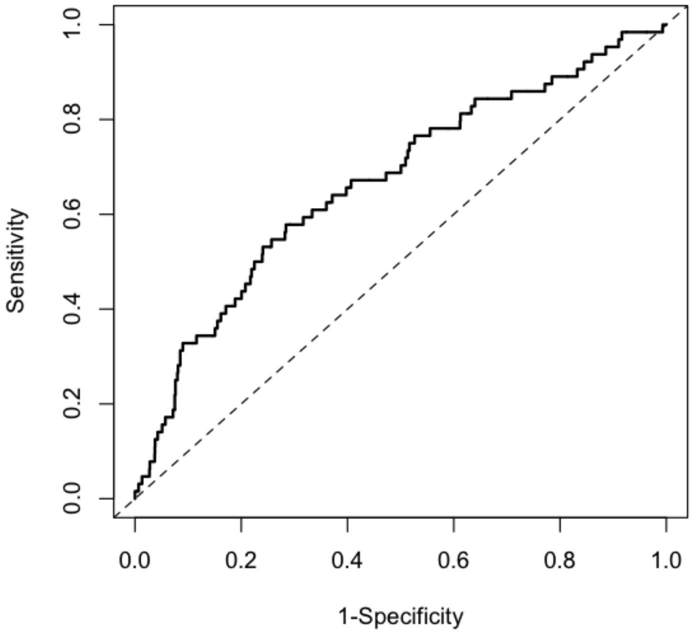

Supplement: Supplementary file 1 — Online Supplemental Material. [file 41598_2020_58652_MOESM1_ESM.pdf]
